# Assessing the health estimation capacity of air pollution exposure prediction models

**DOI:** 10.1186/s12940-022-00844-0

**Published:** 2022-03-17

**Authors:** Jenna R. Krall, Joshua P. Keller, Roger D. Peng

**Affiliations:** 1 Department of Global and Community Health, George Mason University, 4400 University Drive, MS 5B7, Fairfax, VA 22030 USA; 2 Department of Statistics, Colorado State University, 1877 Campus Delivery, Fort Collins, CO 80523 USA; 3 Department of Biostatistics, Johns Hopkins Bloomberg School of Public Health, 615 N Wolfe St, Baltimore, MD 21205 USA

**Keywords:** Ambient air pollution, Chemical transport model, Epidemiology, Exposure assessment, Fourier transform, Health assessment, Particulate matter

## Abstract

**Background:**

The era of big data has enabled sophisticated models to predict air pollution concentrations over space and time. Historically these models have been evaluated using overall metrics that measure how close predictions are to monitoring data. However, overall methods are not designed to distinguish error at timescales most relevant for epidemiologic studies, such as day-to-day errors that impact studies of short-term health associations.

**Methods:**

We introduce frequency band model performance, which quantifies health estimation capacity of air quality prediction models for time series studies of air pollution and health. Frequency band model performance uses a discrete Fourier transform to evaluate prediction models at timescales of interest. We simulated fine particulate matter (PM_2.5_), with errors at timescales varying from acute to seasonal, and health time series data. To compare evaluation approaches, we use correlations and root mean squared error (RMSE). Additionally, we assess health estimation capacity through bias and RMSE in estimated health associations. We apply frequency band model performance to PM_2.5_ predictions at 17 monitors in 8 US cities.

**Results:**

In simulations, frequency band model performance rates predictions better (lower RMSE, higher correlation) when there is no error at a particular timescale (e.g., acute) and worse when error is added to that timescale, compared to overall approaches. Further, frequency band model performance is more strongly associated (*R*^2^ = 0.95) with health association bias compared to overall approaches (*R*^2^ = 0.57). For PM_2.5_ predictions in Salt Lake City, UT, frequency band model performance better identifies acute error that may impact estimated short-term health associations.

**Conclusions:**

For epidemiologic studies, frequency band model performance provides an improvement over existing approaches because it evaluates models at the timescale of interest and is more strongly associated with bias in estimated health associations. Evaluating prediction models at timescales relevant for health studies is critical to determining whether model error will impact estimated health associations.

**Supplementary Information:**

The online version contains supplementary material available at 10.1186/s12940-022-00844-0.

## Background

The United States has for decades monitored air pollution levels via the Environmental Protection Agency’s network of monitors as well as state and local monitors [36]. These monitors tend to be sited around large urban centers or around significant sources of pollution, and as a result, large swaths of the country are typically not monitored and knowledge of air pollution concentrations in those areas has historically been minimal. In the past, one major consequence of this lack of monitoring is that many areas of the country could not be included in studies examining the health associations of air pollutants [2, 39, 44, 55]. The era of big data along with novel machine learning techniques and statistical models have allowed us to predict ambient air pollution concentrations with greater accuracy and precision than in the past [3, 18, 19, 28, 32]. This new generation of models provides air pollution prediction at finer spatial and temporal scales by leveraging multiple sources of data such as satellite data, computer weather models, chemistry models, land use data, emissions source information, and/or pollution monitoring data. These input data sources have varying strengths, for example models may incorporate data from monitors that provide ground truth observations, but have generally limited spatial coverage, and from chemical transport models such as the Community Multiscale Air Quality (CMAQ) model, which have good spatial coverage but are often biased [25, 51]. Predicting air pollution concentrations for epidemiologic applications is challenging and must balance the shortcomings of each input data source.

Given the development of modern prediction models, it is natural to want to evaluate their performance. However, the nature of model evaluation depends critically on the application in which the model will be applied [10, 32]. Without information about study-specific context, it is impossible to provide an unqualified assessment of a model that is informative about the specific application. For example, studies of the acute health associations of ambient air pollution typically focus on day-to-day variation in pollutants and health outcomes [2, 44, 55], suggesting that models predicting pollution concentrations for such studies need to do well predicting the higher frequency temporal components. Studies of chronic health associations of pollution often make broad comparisons across larger geographies [15, 20, 29, 33], suggesting that prediction models there need to reproduce spatial variations in pollution at larger scales, but not finer scale fluctuations.

To evaluate exposure prediction models, metrics such as *R*^2^, root mean square error (RMSE), or normalized root mean square error are commonly used to quantify how predictions vary from ground truth observations [7, 10, 11, 14, 17, 25, 49, 51]. More recent studies have proposed correlation [35] and variance ratios [7], which also capture overall deviations in variability, as being more relevant for evaluating prediction models for use in health studies. Although all these metrics evaluate similarity between model predictions and observations, they do not focus on those errors in prediction models that will most impact estimated health associations. As an example, suppose model predictions correlate well with the observed data at the seasonal and monthly temporal scales, but correlations are low at the day-to-day scale. Metrics such as *R*^2^ are impacted by performance at all temporal scales and will not highlight poor performance at the day-to-day scale most relevant for acute epidemiologic studies. Conversely, for a model that performs well primarily at the day-to-day scale, these metrics may be overly pessimistic for model performance in an acute epidemiologic study.

Existing methods for *evaluating* prediction models do not incorporate temporal and spatial scales of interest. This presents a gap in the use of novel methods, such as machine learning approaches, for exposure estimation in epidemiologic studies. Therefore, the objective of the present research is to propose a new approach to evaluate prediction models that focuses on temporal scales that will most impact estimated health associations. We demonstrate how usual model performance metrics such as RMSE can fail to capture errors in prediction models that are relevant to epidemiologic studies. Additionally, we propose frequency band model evaluation to determine whether a given prediction model will provide good estimates of health associations. Our approach evaluates the health estimation capacity of prediction models by focusing model evaluation on the timescale of interest of the health effect. We illustrate model evaluation using particulate matter air pollution less than 2.5 *μ* m in aerodynamic diameter (PM_2.5_), though our methods are applicable to prediction models of air pollutants generally (e.g., ozone and nitrogen dioxide (NO_2_)).

## Methods

We observe a time series of ground-truth observations *z*(*t*), which may represent air pollution measurements (e.g., for PM_2.5_) taken at a central site monitor. The goal of an exposure prediction model is to accurately replicate such observations with a predicted time series *z*^∗^(*t*) that can be computed at locations and times without monitoring data. The predicted series *z*^∗^(*t*) may represent output from a regression model, a machine learning algorithm, a computer simulation model such as the CMAQ model, or any combination of these approaches. While we will focus on temporal data series *z*(*t*) and predictions *z*^∗^(*t*) indexed by time *t*, we discuss extensions of these ideas to the spatial domain in the Discussion.

Our goal is to compare a time series of model predictions, *z*^∗^(*t*), with a reference time series of ground-truth observations, *z*(*t*), for times *t* = 1, …, *n*. Existing approaches include quantifying prediction accuracy using correlation $r=\sqrt{R^2}=\mathrm{Cor}\left({z}^{\ast }(t),z(t)\right)$ and $RMSE=\sqrt{\frac{1}{n}\sum_{t=1}^n{\left({z}^{\ast }(t)-z(t)\right)}^2}$ [7, 11, 14, 35]. Additional existing approaches include the log variance ratio (*LVR*), defined as $\log \left(\frac{\mathrm{Var}\left({z}^{\ast }(t)\right)}{\mathrm{Var}\left(z(t)\right)}\right)$ [7]. The *LVR* captures differences in temporal variation between models, which can impact precision in estimated health associations. We refer to these approaches as *overall model performance* measures, which we denote by *r*_overall_, *RMSE*_overall_, and *LVR*_overall_.

Overall model performance can be impacted by model errors at timescales different than the timescale of interest. Therefore to better capture health estimation capacity, we propose *frequency band model performance.* Frequency band model performance differs from existing approaches by restricting the model predictions *z*^∗^(*t*) and observations *z*(*t*) to their timescale-specific components to create measures *r*_(*k*)_, *RMSE*_(*k*)_, and *LVR*_(*k*)_, which represent correlation, RMSE, and LVR for a chosen range of frequencies denoted as band *k*. To extract the frequency band *k* components, we use a discrete Fourier transform [6, 23, 40]. For our reference time series, *z*(*t*), we partition the range of frequencies [0, *n*/2) into non-overlapping frequency bands *k* = 1, …, *K* such that

1 $$z(t)=\sum_{k=1}^K{z}_k(t)$$

The same approach is used to partition *z*^∗^(*t*) into components ${z}_k^{\ast }(t)$. Then, ${r}_{(k)}=\mathrm{Cor}\left({z}_k^{\ast }(t),{z}_k(t)\right)$, $RMS{E}_{(k)}=\sqrt{\frac{1}{n}\sum_{t=1}^n{\left({z}_k^{\ast }(t)-{z}_k(t)\right)}^2}$, and $LV{R}_{(k)}=\log \left(\frac{\mathrm{Var}\left({z}_k^{\ast }(t)\right)}{\mathrm{Var}\left({z}_k\right)\Big)}\right)$. To facilitate comparisons between overall and frequency band *k* RMSE, we scale *RMSE*_overall_ and *RMSE*_(*k*)_ by the standard deviation of the reference time series *z*(*t*) and *z*_*k*_(*t*), respectively.

**Fig. 1:**
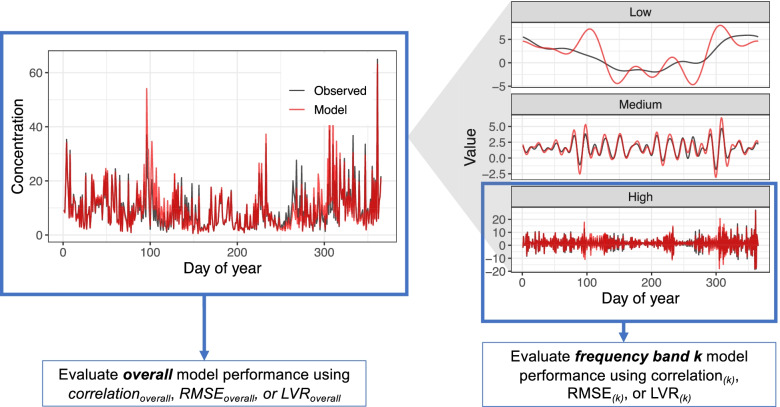
Hypothetical example of overall and frequency band model evaluation. The existing approach of overall model performance is impacted by errors in time series at all frequencies (e.g., high, medium, and low), whereas our frequency band model evaluation quantifies errors in the relevant frequency (e.g., high frequency extracted using discrete Fourier transform) for evaluating prediction models in acute health studies

To understand the advantage of frequency band model evaluation, consider a hypothetical time series of PM_2.5_ observations and model predictions (Fig. 1). Existing overall model performance metrics (*r*_overall_, *RMSE*_overall_, and *LVR*_overall_) are applied to the time series on the left, which is impacted by differences between observations and model predictions at all frequencies. In contrast, frequency band *k* model performance restricts evaluation of model performance to the frequency of interest using a discrete Fourier transform (e.g., high frequency, such as day-to-day variation) and is not impacted by errors at other frequencies (e.g., medium frequency, such as monthly variation, and low frequency, such as seasonal variation) (Fig. 1).

As in previous work estimating health associations of PM air pollution [23], we set *K* = 6 and consider frequency bands of *k* = 1: [1,6) cycles per year corresponding to seasonal components, *k* = 2: [6,12) cycles per year, *k* = 3: [12,26) cycles per year, *k* = 4: [26,52) cycles per year, *k* = 5: [52,104) cycles per year, and *k* = 6: [104,183) cycles per year corresponding to acute components. Our particular interest for acute health associations of air pollution is in the acute timescale captured by the frequency band *k* = 6 model performance (*k* = 6: [104,183) cycles per year), corresponding to a timescale of a few days or less. However, frequency band model performance can be applied to any timescales that are of interest.

We first conduct a simulation study to determine how errors at specific frequency bands impact overall model performance measures *r*_overall_, *RMSE*_overall_, and *LVR*_overall_ as well as frequency band *k* model performance measures *r*_(*k*)_, *RMSE*_(*k*)_, and *LVR*_(*k*)_. We hypothesize that errors at a specific frequency band *k*′ impact the *overall* model performance (*r*_overall_, *RMSE*_overall_, and *LVR*_overall_) and the model performance at the same frequency band *k*′ (*r*_(*k*′)_, *RMSE*_(*k*′)_, and *LVR*_(*k*′)_), but not model performance at different frequency bands *k* ≠ *k*′. For example, seasonal error that occurs within the first frequency band (*k* = 1 corresponding to [1,6) cycles per year) would impact model performance measured by *r*_overall_, *RMSE*_overall_, *LVR*_overall_, *r*_(1)_, *RMSE*_(1)_, and *LVR*_(1)_, but not model performance at higher frequency bands relevant for estimating acute health associations (e.g., *r*_(6)_, *RMSE*_(6)_, and *LVR*_(6)_). This implies that if we are primarily interested in model predictions at frequency band *k*′, model performance measured by *r*_(*k*′)_, *RMSE*_(*k*′)_, and *LVR*_(*k*′)_ will better reflect errors relevant for frequency band *k*′ while limiting the influence of errors at other frequency bands *k* ≠ *k*′.

To simulate observed air pollution time series that are non-negative and right-skewed, let *z*(*t*) = exp(*x*(*t*)*σ*_*x*_ + *μ*_*x*_), where *x*(*t*) is a time series with Var(*x*(*t*)) = 1, and where *μ*_*x*_ and *σ*_*x*_ represent the mean and standard deviation of the log-transformed time series. We specify *μ*_*x*_ = 1.9 log *μ* g/m^3^ and *σ*_*x*_ = 0.6 log *μ* g/m^3^ to reflect log-transformed concentrations of PM_2.5_ in New York City from 2010 to 2018. We simulate the time series $x(t)=\frac{\sum_{k\in \left\{1,2,6\right\}}{x}_k(t)}{\sqrt{\mathrm{Var}\left(\sum_{k\in \left\{1,2,6\right\}}{x}_k(t)\right)}}$ consisting of frequency bands *k* = 1, 2, 6 to approximately reflect seasonal, monthly, and acute time trends found in air pollution concentrations. Each *x*_*k*_(*t*) is specified using cosine functions with varying wavelengths (details in Additional file, Section A) and Var(*x*_*k*_(*t*)) = 1. As a sensitivity analysis, we increase the relative seasonal variation to reflect observed relative variability across timescales in PM_2.5_ data, i.e. Var(*x*_(1)_(*t*)) ∈ {1.5,2}. We simulate 100 observed time series with 3 years of data each (*n* = 1095).

**Fig. 2:**
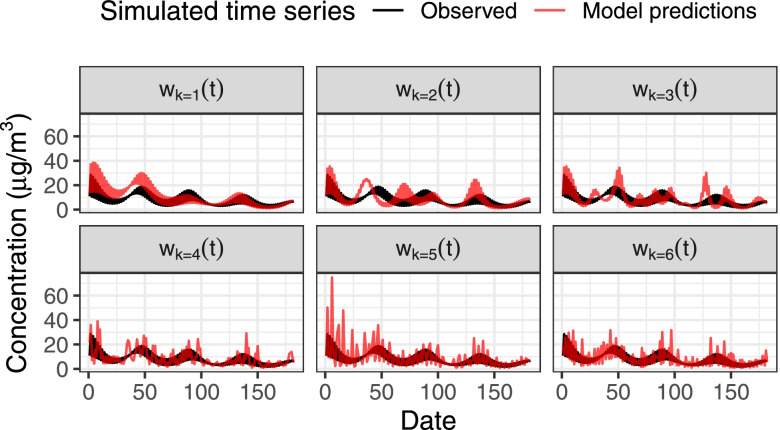
Example simulated observed and predicted PM_2.5_ time series. Observed time series and model predictions are shown magnitude of error *σ*_*c*_ = 0.8 and frequency band classical error *w*_*k*_(*t*) for *k* = 1, …, 6

We simulate model predictions by incorporating classical measurement error at varying timescales into simulated observed time series. Our model predictions are simulated as *z*^∗^(*t*) = exp(*x*^∗^(*t*)*σ*_*x*_ + *μ*_*x*_), with *μ*_*x*_ = 1.9 and *σ*_*x*_ = 0.6 as in the simulated observed time series. The log-transformed simulated model predictions *x*^∗^(*t*) = *x*(*t*) + *w*_*k*_(*t*)*σ*_*c*_, where *x*(*t*) is the simulated observed time series (log-transformed with Var(*x*(*t*)) = 1) and *w*_*k*_(*t*) is the standardized error component at frequency band *k* with Var(*w*_*k*_(*t*)) = 1, The magnitude of error is represented by the standard deviation *σ*_*c*_ ∈ {0.2,0.4,0.6,0.8}. We obtain *w*_*k*_(*t*) as the standardized *k* frequency band component from a discrete Fourier transform of standard normal error. Therefore, on the logarithmic scale, our simulated model predictions are the simulated observed time series with classical error at frequency band *k*. As *k* varies from 1, …, 6, the timescale of the error changes from seasonal (*k* = 1) to acute (*k* = 6) (Fig. 2). For each of our 100 simulated observed time series with *n* = 1095, we simulate 24 model prediction time series with varying classical measurement error (four varying magnitudes of error *σ*_*c*_ and six error frequencies *w*_*k*_(*t*) *k* = 1, …, 6).

Health counts (e.g., number of deaths or emergency department (ED) visits for day *t*) are simulated as Poisson(*μ*(*t*)) where *μ*(*t*) = exp(*β*_0_ + *β*_1_*z*^residual^(*t*) + 0.03*z*^fitted^(*t*)). The residuals *z*^residual^(*t*) and fitted *z*^fitted^(*t*) are obtained from a regression of the simulated observed time series *z*(*t*) on a natural spline of time with 24 degrees of freedom to capture seasonal and monthly trends. Therefore, the residuals *z*^residual^(*t*) represent the sub-monthly frequency components of *z*(*t*) relevant for acute health associations. We specify the acute health association *β*_1_ = log(1.1)/10 corresponding to a relative risk of 1.1 per 10 *μ* g/m^3^ increase in PM_2.5_. The base rate of health *β*_0_ = 5 yields approximately 200 health counts per day (i.e., deaths or ED visits) and is selected to reflect cardiorespiratory ED visits observed in large U.S. cities [34].

We estimate health associations for simulated model predictions with varying magnitudes of classical error (*σ*_*c*_) and error *w*_*k*_(*t*) at frequencies 1, …, 6. The health association model is an overdispersed Poisson time series regression model controlling for non-acute temporal trends with a natural spline of time with 280 degrees of freedom per year to isolate the acute time series. This number of degrees of freedom is much larger than generally used in the epidemiologic literature to properly adjust for *w*_*k*_(*t*) error added at *k* = 5 [52, 104) cycles per year. In practice, confounding in epidemiologic studies of acute health associations is controlled for by both a smooth function of time (generally with <10 degrees of freedom per year) as well as smooth functions for meteorology including temperature and humidity [37, 38, 44, 45, 50]. To evaluate the estimated health associations for the model predictions, we compute the estimated health association RMSE and percent relative mean bias in the estimated health association (mean bias/*β*_1_ × 100). Using scatterplots and *R*^2^, we examine associations of both mean overall model performance and frequency band *k* model performance with health association RMSE and percent relative mean bias across 100 simulated datasets.

We also compare observations of PM_2.5_ to predictions from an exposure model to demonstrate both overall model performance and frequency band *k* = 6 model performance. To represent “ground-truth” observations, we develop a dataset of observed daily PM_2.5_ data in *μ* g/m^3^ from 17 monitors across 8 US cities from 2010 to 2017 using the US Environmental Protection Agency’s (US EPA) Air Quality System. We select monitors from cities based on geographic locations throughout the continental US and based on previous studies of air pollution and health [23, 34, 44, 55], including Atlanta, GA (number of monitoring sites: *n* = 4); Dallas, TX (*n* = 1); Houston, TX (*n* = 1); Los Angeles, CA (*n* = 5); New York City, NY (*n* = 1); Pittsburgh, PA (*n* = 2); Seattle/Tacoma, WA (*n* = 1); and Salt Lake City, UT (*n* = 2). For each monitor, we interpolate PM_2.5_ means for short gaps (<10 days) in the data for each city to create uninterrupted time series. We include only monitors that had at least 1 year of daily concentrations after interpolation. We utilize the longest complete time series for each monitor.

As our prediction model, we utilize predictions from the Fused Air Quality Surface Using Downscaling (FAQSD) approach at 2010 US census tracts from the US EPA [52]. FAQSD uses a Bayesian space-time model to fuse monitoring data with CMAQ model predictions and develop predictions at 2010 US census tracts [3–5, 42]. CMAQ is an atmospheric chemical transport model that provides predictions 12 × 12 km resolution grids across the US [12, 13] and may be calibrated or fused with observed monitoring data [21]. We link FAQSD predictions to monitors using the census tract where the monitor is located. We compare the observed PM_2.5_ data and FAQSD using overall performance measures (*r*_overall_, *RMSE*_overall_, and *LVR*_overall_) and frequency band *k* = 6 model performance (*r*_(6)_, *RMSE*_(6)_, and *LVR*_(6)_), where *k* = 6 corresponds to [104,183) cycles per year and represents variation relevant for acute health associations at timescales <3.5 days [23]. All analyses were conducted using R version 4.0 [41].

## Results

**Fig. 3:**
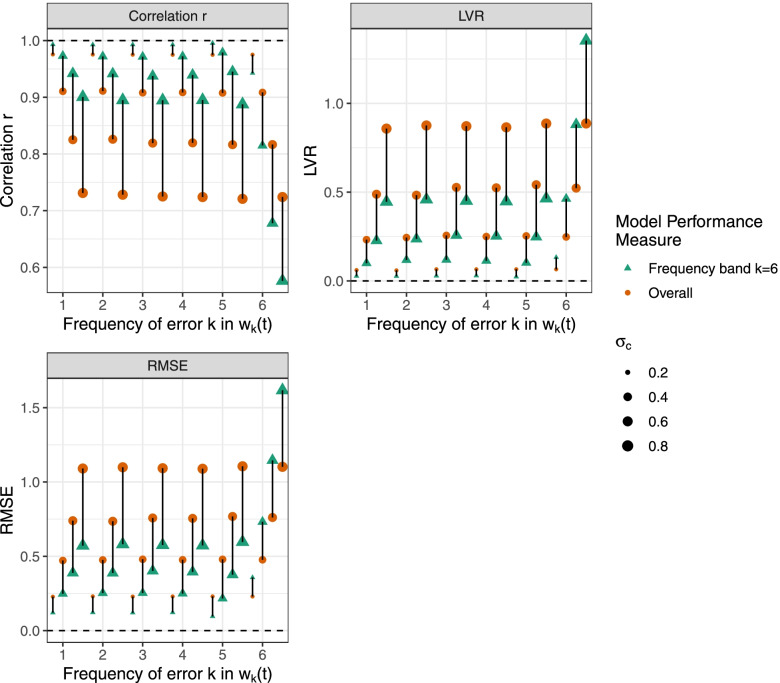
Comparison of simulated time series and model predictions for overall and frequency band model performance. Results are shown using overall model performance (orange circles) and frequency band *k* = 6 model performance (green triangles) for correlation *r*, *RMSE*, and *LVR*. For model predictions, classical error *w*_*k*_(*t*) was added to simulated observations at frequency bands *k* = 1, …, 6 with magnitude of error *σ*_*c*_ = {0.2,0.4,0.6,0.8}

In our simulation study, we evaluate model performance by comparing *z*(*t*) and *z*^∗^(*t*) using overall model performance (*r*_overall_, *RMSE*_overall_, and *LVR*_overall_) and frequency band *k* model performance *r*_(*k*)_, *RMSE*_(*k*)_, and *LVR*_(*k*)_. We focus on frequency band *k* = 6 model performance, (*r*_(6)_, *RMSE*_(6)_, and *LVR*_(6)_), which evaluates the high frequency of the component of the model and is most relevant for estimating acute health associations. Fig. 3 shows the mean across 100 simulated datasets for overall model performance measures (orange circles) and frequency band *k* = 6 model performance measures (green triangles) for error *w*_*k*_(*t*), *k* = 1, …, 6. With error *w*_*k*_(*t*) at frequency bands *k* = 1, …, 5, the frequency band *k* = 6 model performance (*r*_(6)_, *RMSE*_(6)_, and *LVR*_(6)_) rated the prediction model better compared to the overall model performance (*r*_overall_, *RMSE*_overall_, and *LVR*_overall_). When error is added using the high frequency band *k* = 6, i.e., component *w*_(6)_(*t*), the frequency band *k* = 6 model performance rated the prediction model worse compared to the overall model performance. The results are consistent across model performance measures of *r*, *RMSE* and *LVR*. Further, the results are consistent for frequency band *k* = 1, 2 model performance (Additional file, Fig. S1), with frequency band *k* model performance better reflecting errors at frequency band *k*. We did not examine frequency band *k* = 3, …, 5 model performance because the simulated observed time series did not have variation at those frequencies. In summary, overall model performance can be both overly pessimistic when the prediction model has error at timescales not relevant to the study design and overly optimistic when the prediction model has error at relevant timescales. Frequency band *k* model performance better reflects error at timescales of interest.

As a sensitivity analysis, we increase the relative variance of the seasonal component of the simulated observed time series Var(*x*_(1)_(*t*)) ∈ {1^2^, 1.5^2^, 2^2^} while keeping Var(*x*_(2)_(*t*)) = Var(*x*_(6)_(*t*)) = 1 and the total variance Var(*x*(*t*)) = 1 to reflect observed timescale variability in PM_2.5_ concentrations. Changing the variance of the seasonal component alone did not impact overall model performance. However the frequency band *k* = 6 model performance rates the models worse with increasing Var(*x*_(1)_(*t*)) (Additional file, Fig. S2). Because the entire time series is scaled such that Var(*x*(*t*)) = 1, the acute component of the simulated time series has decreasing variance as Var(*x*_(1)_(*t*)) increases. In other words, holding the total variability of the time series constant, the same magnitude of error *σ*_*c*_ has a stronger impact on the acute component of the time series when the acute component has lower relative variance.

**Fig. 4:**
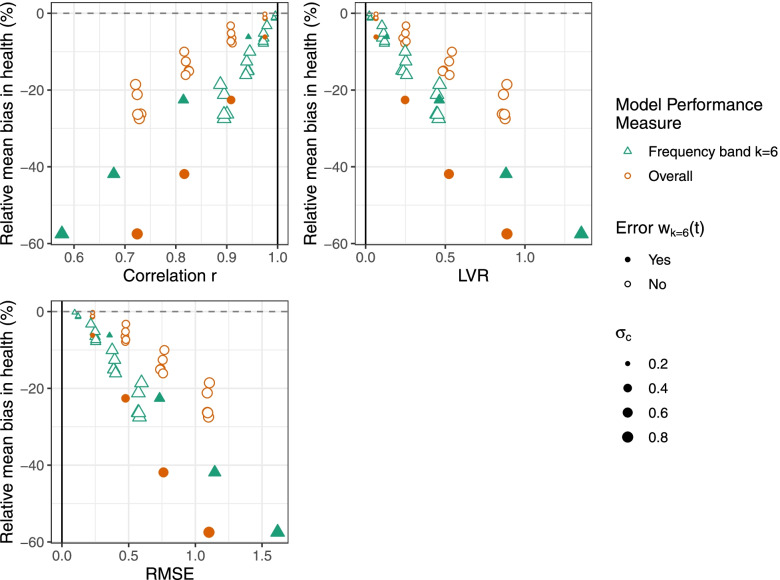
Associations of percent relative mean bias in estimated health associations with model performance. Results are shown using overall model performance (orange circles) and frequency band *k* = 6 model performance (green triangles) for correlation *r*, *RMSE*, and *LVR*. For model predictions, classical error *w*_*k*_(*t*) was added to simulated observations at frequency bands *k* = 1, …, 6 (acute error *w*_*k* = 6_(*t*) is shaded) with magnitude of error *σ*_*c*_ = {0.2,0.4,0.6,0.8}

**Fig. 5:**
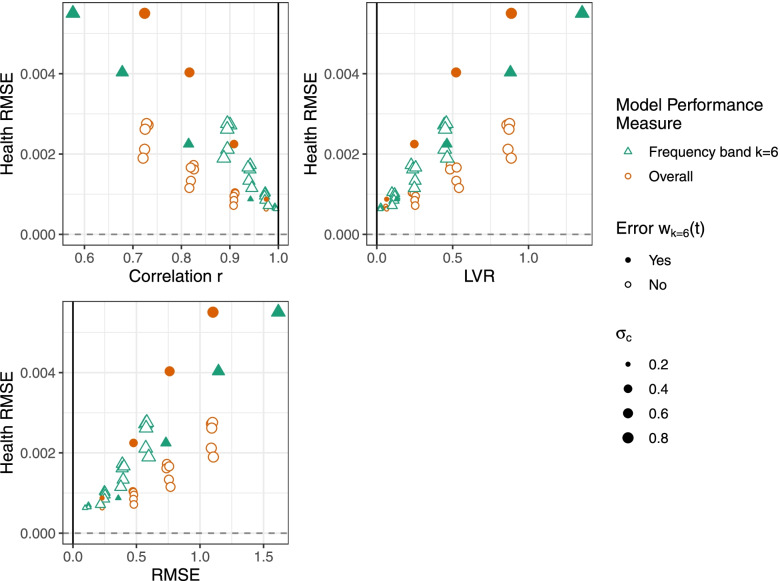
Associations of health association RMSE with model performance . Results are shown using model performance (orange circles) and frequency band *k* = 6 model performance (green triangles) for correlation *r*, *RMSE*, and *LVR*. For model predictions, classical error *w*_*k*_(*t*) was added to simulated observations at frequency bands *k* = 1, …, 6 (acute error *w*_*k* = 6_(*t*) is shaded) with magnitude of error *σ*_*c*_ = {0.2,0.4,0.6,0.8}

To determine whether overall model performance or frequency band *k* model performance better capture health estimation capacity, we examine associations of model performance measures (*r*, *RMSE*, *LVR*) with bias in estimated health associations as well as estimated health association RMSE. We focus on frequency band *k* = 6 model performance that evaluates the high frequency component of the time series relevant for estimating *acute* health associations. We expect larger bias and RMSE in estimated acute health associations for model predictions with high frequency error *w*_*k* = 6_(*t*). Figure 4 shows the percent relative mean bias against the mean overall model performance (orange circles) and the mean frequency band *k* = 6 model performance (green triangles). The solid points indicate when acute frequency band error *w*_(6)_(*t*) is added and therefore expected to strongly impact bias in the acute health association, and outlined otherwise. The size of the point indicates the magnitude of error added. For the frequency band *k* = 6 model performance, *r*_(6)_, *RMSE*_(6)_, and *LVR*_(6)_ are more strongly associated with bias compared to the overall performance measures (*r*_overall_, *RMSE*_overall_, and *LVR*_overall_). Similarly, the association between frequency band *k* = 6 model performance and health RMSE is also stronger compared to the overall performance (Fig. 5). The linear association *R*^2^ with acute health association measures (percent relative mean bias and health RMSE) is stronger for frequency band *k* = 6 model performance compared to overall model performance (Table 1). For example, percent relative mean bias was more strongly associated with frequency band *k* = 6 *RMSE*_(6)_ (*R*^2^ = 0.95) compared to overall *RMSE*_overall_ (*R*^2^ = 0.57).

**Table 1:** *R*^2^ for the linear relationship of exposure metrics with health measures

Health measure	Metric	Frequency band k = 6	Overall
Rel. mean bias (%)	Correlation r	0.89	0.57
	LVR	0.96	0.58
	RMSE	0.95	0.57
Health RMSE	Correlation r	0.90	0.52
	LVR	0.96	0.53
	RMSE	0.94	0.52

**Fig. 6:**
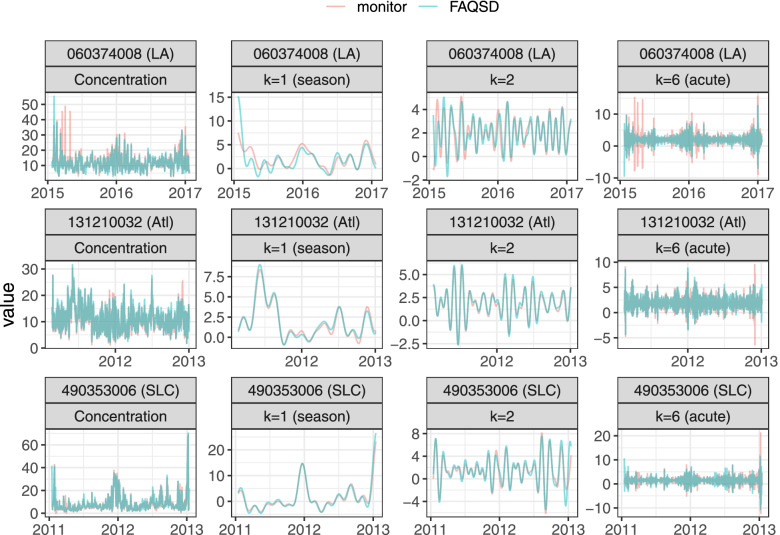
Daily PM_2.5_ concentrations and model predictions using FAQSD for three U.S. monitors. Results are shown for the overall time series and the decomposed time series at *k* = 1 (season), *k* = 2, and *k* = 6 (acute) frequency bands for the first 2 years for 3 example monitors: 060374008 (Los Angeles), 131210032 (Atlanta), 490353006 (Salt Lake City)

For the analysis of PM_2.5_ predictions, the locations of the selected PM_2.5_ monitors were spread across the US (Additional file, Fig. S3). The available daily observations range from 593 days (monitor site 420030008 in Pittsburgh) to 2351 days (monitor site 360810124 in New York City) (Additional file, Table S1). The lowest median PM_2.5_ concentration is in Seattle/Tacoma (5.4 *μ* g/m^3^) and the highest in Los Angeles (12 *μ* g/m^3^). Figure 6 compares the overall concentration time series and three frequency band components from a discrete Fourier transform (Eq. 1): *k* = 1 or the seasonal component, *k* = 2 or the monthly component, and *k* = 6 the acute component. The monitors include 060374008 in Los Angeles where FAQSD and the monitor differ considerably at all timescales, 131210032 in Atlanta where FAQSD and the monitor are similar at all timescales, and 490353006 in Salt Lake City where FAQSD performs similarly to the monitor at longer timescales (monthly, seasonal), but not at shorter timescales (acute).

**Fig. 7:**
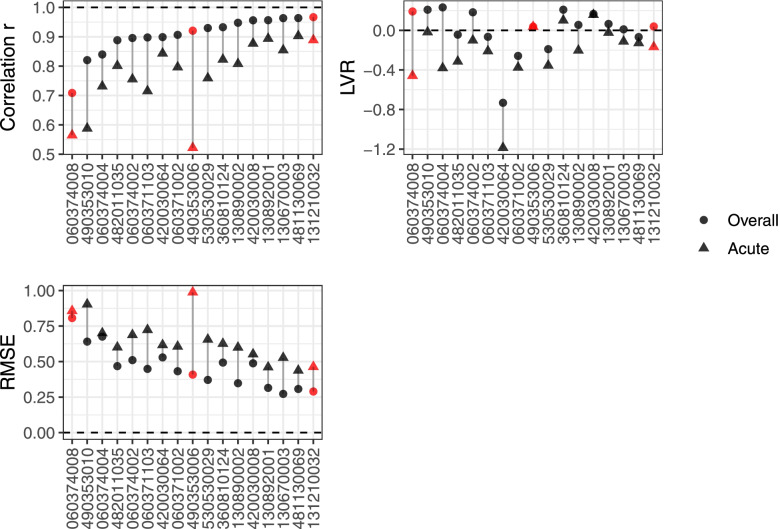
Comparing observed PM_2.5_ data and FAQSD model predictions for 17 U.S. monitors. Results are shown using overall model performance (circles) and frequency band *k* = 6 model performance (triangles) for correlation *r*, *RMSE*, and *LVR* for 17 monitors. Points in red indicate example monitors of 060374008 (Los Angeles), 131210032 (Atlanta), and 490353006 (Salt Lake City)

Comparing overall performance and frequency band *k* = 6 model performance of FAQSD for all 17 monitors, the overall performance measures rate FAQSD better compared to frequency band *k* = 6 model performance (Fig. 7). The three example monitors are shown in red. For overall model performance, correlations below *r* = 0.89 (*R*^2^ = 0.8) are considered low. For 060374008 in Los Angeles, both the overall and frequency band *k* = 6 measures rate FAQSD low (*r* = 0.71 and *r* = 0.56, respectively). For 131210032 in Atlanta, both the overall and frequency band *k* = 6 model performance measures rate FAQSD well (*r* = 0.97 and *r* = 0.89, respectively). However, at 490353006 in Salt Lake City, there is a large discrepancy between the overall and frequency band *k* = 6 correlation and RMSE, where the performance of FAQSD may be overrated using the overall approach and may be overly optimistic about its acute health estimation capacity (e.g., *r* = 0.92 and *r* = 0.52, respectively). This is likely driven by the good performance of FAQSD at this monitor at the seasonal timescale, but not at the acute timescale (Fig. 6).

## Discussion

We propose frequency band model performance for evaluating health estimation capacity of air quality prediction models. When comparing model predictions to truth in simulations, frequency band *k* correlation *r*_(*k*)_, *RMSE*_(*k*)_, and *LVR*_(*k*)_ better reflect error at specific timescales compared to overall metrics. Of particular relevance to acute epidemiologic studies, frequency band *k* = 6 model performance penalizes models for error at acute timescales, with lower correlation *r* and higher RMSE, while reporting higher model performance when error is not present at the acute timescale. Furthermore in simulations of estimated acute health associations, frequency band *k* = 6 model performance is more strongly associated with relative mean bias and RMSE in estimated health associations. In a study of 8 US cities, overall model performance and frequency band *k* = 6 model performance can rate prediction models differently, emphasizing the need for a model performance tool that is best suited to the proposed analysis.

Recent studies have evaluated or compared the performance of air quality prediction models [11, 14, 32, 35]. Although many previous studies used primarily overall *RMSE* and correlation (*r*) for model performance [7, 11, 14, 35], *LVR* can capture differences relevant for precision of estimated health associations [7]. Whether overall or frequency band model performance is used, examining multiple metrics such as *r*, *RMSE*, and *LVR*, can help elucidate different aspects of model performance. Further, while we demonstrate frequency band model evaluation using PM_2.5_, our approach can be directly applied to evaluate prediction models for other pollutants examined in previous studies such as NO_2_ [7, 11, 14, 35] and ozone [7, 35].

Effects of measurement error in time series studies of air pollution and health has been extensively examined using simulation studies [7, 8, 22, 27, 48]. Studies have examined spatial errors [48], error type [27], as well as effects of measurement error in multipollutant models [22] and multi-level models [7, 8]. Our work adds to this literature by simulating measurement error at varying timescales. This can better reflect practice where a prediction model may have errors in the seasonal component, but not the acute component, or vice versa.

Previous epidemiologic studies have used timescale decomposition approaches to determine health associations of air pollution at varying timescales [23, 46]. As in previous work [23], we decompose the time series into components corresponding to different timescales using a discrete Fourier transform [6, 40]. More recent epidemiologic studies utilized distributed lag models to estimate health associations at varying timescales [26, 47, 53, 54]. For analyses of health associations at different time scales, frequency band model performance can be applied in the planning stage of an analysis before health data are collected to evaluate whether a prediction model can be effectively used for the proposed timescales of interest.

Aside from epidemiologic studies, there are analyses for which overall model performance will be more appropriate compared to frequency band model performance. For analyses estimating burden of disease due to air pollution, representing the true concentrations, and not short or long-scale variability, is most important [24, 30]. Furthermore, both frequency band and overall model performance represent “operational evaluation” of the model biases [16] that describe deviations of the predictions from the truth. Depending on use, air quality prediction models should be evaluated with respect to multiple performance features, including operational evaluation, “diagnostic evaluation” of whether model errors are driven by inputs, and other features [16]. A recent study of CMAQ, along with other air quality models and statistical modeling approaches, did not find substantial differences in regional and national scale spatial PM_2.5_ predictions from these models, but the authors note that the best model may vary with how the model will be used [32].

We incorporated classical measurement error in our simulation study. Additive classical measurement error biases estimated health associations [9, 56]. In practice, the error may be a more complex combination of both Berkson and classical error [7]. Additive error on the log scale, as we simulated in this work, can introduce bias in estimated health associations when the error type is classical or Berkson [27]. The goal of the present study was to demonstrate that error at varying timescales was better captured by frequency band model performance compared to overall model performance, and not to examine measurement error types.

Frequency band model performance can be directly applied to time series studies of health associations, such as studies of acute health associations. Future work could extend our method to epidemiologic studies of long-term exposure to air pollution and health. Extensions of frequency band model evaluation to long-term epidemiologic studies will need to address several challenges. Assessing effects of long-term exposure on health relies on accurately representing spatial contrasts in concentrations across different locations. The natural extension of frequency band model performance to a spatial setting would require applying a two-dimensional Fourier transformation, which necessitates gridded data. Prediction model output is often available over spatial grids [18, 43], and a recent study estimated associations of PM_2.5_ and birthweight at varying spatial scales using wavelet decomposition [1]. Similarly, an assessment of unmeasured spatial confounding at different scales compared Fourier and wavelet decompositions of exposure [31]. However, the spatial distribution of monitoring locations do not follow a regular grid, limiting the application of the frequency band approach to these contexts. Increasing the spatial density of the monitoring network, perhaps through the use of low-cost monitors, could mitigate this issue and allow for decomposition of the spatial scales of variation in monitoring data.

## Conclusions

Frequency band model performance can be applied to predictions from air quality models to evaluate performance at timescales of interest for epidemiologic studies. Compared to commonly-used overall model evaluation approaches, frequency band model evaluation better reflects error at timescales of interest and is more strongly associated with bias in estimated health associations. Multiple metrics should be used to evaluate the performance of air quality models. When the model predictions will be used for health analyses, it is important to evaluate the model performance at timescales that will impact the estimated health associations.

## Supplementary Information

**Additional file 1.** Methods for simulation study and additional figures and tables.

## Data Availability

The datasets and code for the data analysis are available as an R package on GitHub [https://github.com/kralljr/tsfreqband].

## Abbreviations

CMAQ: Community multiscale air quality model
RMSE: Root mean square error
PM_2.5_: Particulate matter less than 2.5 *μ* m
NO_2_: Nitrogen dioxide
LVR: Log variance ratio
ED: Emergency department
FAQSD: Fused air quality surface using downscaling
