# Supplementary material for: Assessing the health estimation capacity of air pollution exposure prediction models
Source: Environ Health. 2022 Mar 17;21:35. doi: 10.1186/s12940-022-00844-0 (PMC8928613; doi:10.1186/s12940-022-00844-0)
Supplement: Supplementary file 1 — Additional file 1. Methods for simulation study and additional figures and tables. [file 12940_2022_844_MOESM1_ESM.pdf]

# Assessing the health estimation capacity of air pollution exposure prediction models

Additional file

## Contents

**Section A. Simulated seasonal, monthly, and acute time trends** **1**

**Figure S1. Comparing simulated observed time series and model predictions using overall model performance (orange circles) and frequency band $k$ model performance (triangles) for correlation $r$, $RMSE$, and $LVR$. For model predictions, classical error $w^k(t)$ was added to simulated observations at frequency bands $k = 1, \ldots, 6$ with magnitude of error $\sigma_c = \{0.2, 0.4, 0.6, 0.8\}$. Results are shown for frequency band k ($k = 1, 2$) model performance.** **2**

Figure S1a. Frequency band $k = 1$ model performance (purple triangles) . . . . . . . . . . . . . . . 2

Figure S1b. Frequency band $k = 2$ model performance (pink triangles) . . . . . . . . . . . . . . . . . 3

**Figure S2. Comparing simulated observed time series and model predictions using overall model performance (orange circles) and frequency band $k = 6$ model performance (green triangles) for correlation $r$, $RMSE$, and $LVR$ with magnitude of error $\sigma_c = \{0.2, 0.4, 0.6, 0.8\}$. This plot shows the impact of changing the variance of the seasonal component of the simulated observed time series, Var($x_{(1)}(t)$) $\in \{1^2, 1.5^2, 2^2\}$.** **4**

**Figure S3. Map of 17 $PM_{2.5}$ monitors across 8 US cities.** **5**

**Table S1. For each monitor ID, table of the corresponding city and state, number of days of data, number and percent of imputed monitor observations, and median (interquartile range) $PM_{2.5}$ ($\mu$g/m$^3$) for both the monitoring data and FAQSD.** **5**

**References** **6**

## Section A. Simulated seasonal, monthly, and acute time trends

To create timescale components for $k = 1, 2, 6$, we specified $x^k(t) = \frac{\tilde{x}^k(t)}{\text{Var}(\tilde{x}^k(t))}$ where

$$\tilde{x}^k(t) = cos\left(\frac{2\pi\gamma}{365} \times t\right)$$

corresponding to $\gamma$ cycles per year. For the seasonal trend ($k = 1$), we set $\gamma = 1$ such that one complete cycle was completed each year. For the monthly ($k = 2$), we set $\gamma = 8$, which fits within the second frequency band of [6-12) cycles per year from Dominici et al. 2003 (Dominici et al. 2003). Last, for the acute trend ($k = 6$), we specified $\gamma = 180$, or approximately a cycle every two days.

**Figure S1. Comparing simulated observed time series and model predictions using overall model performance (orange circles) and frequency band $k$ model performance (triangles) for correlation $r$, $RMSE$, and $LVR$. For model predictions, classical error $w^k(t)$ was added to simulated observations at frequency bands $k = 1, \ldots, 6$ with magnitude of error $\sigma_c = \{0.2, 0.4, 0.6, 0.8\}$. Results are shown for frequency band k ($k = 1, 2$) model performance.**

**Figure S1a. Frequency band $k = 1$ model performance (purple triangles)**

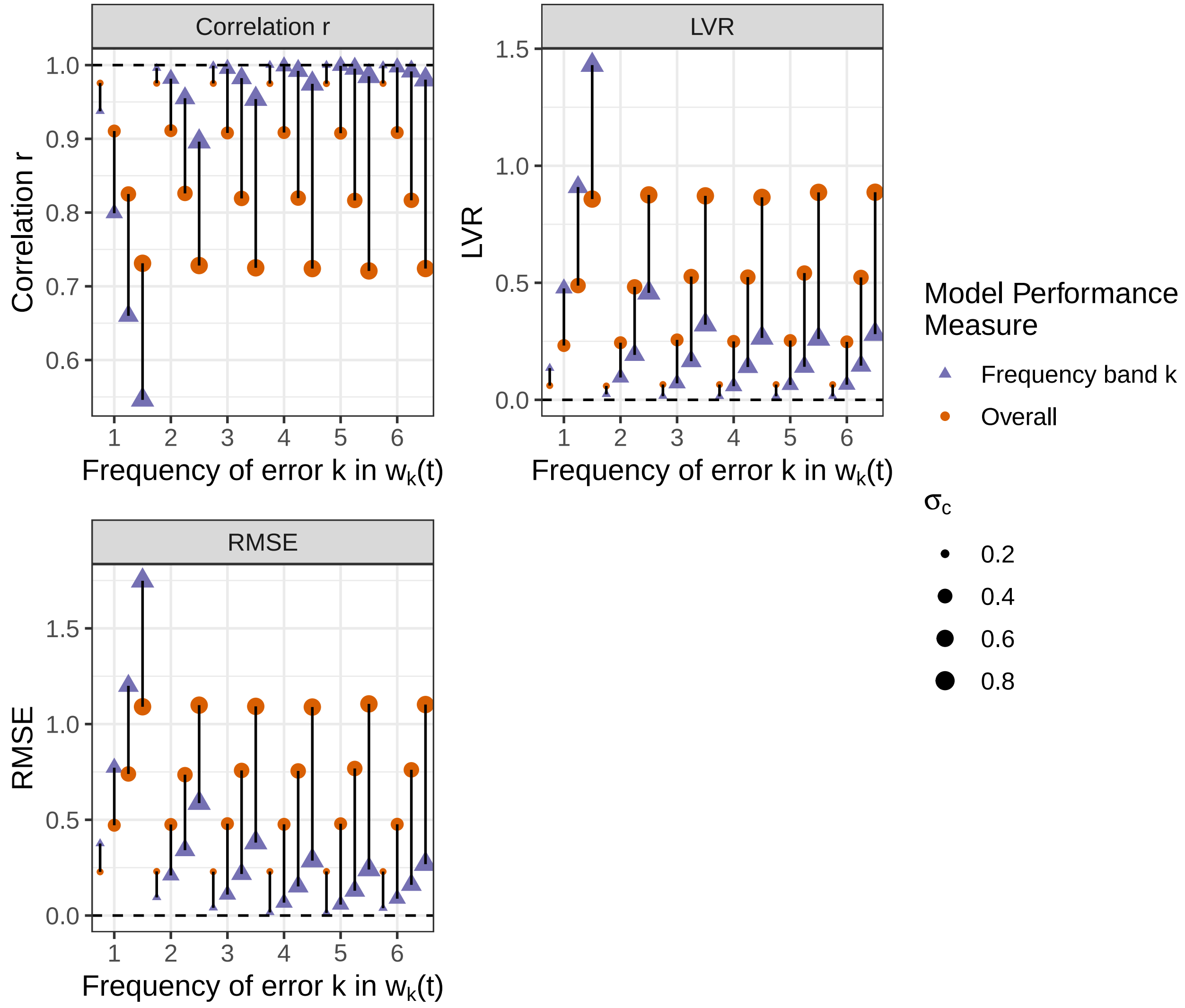

**Figure S1b. Frequency band $k = 2$ model performance (pink triangles)**

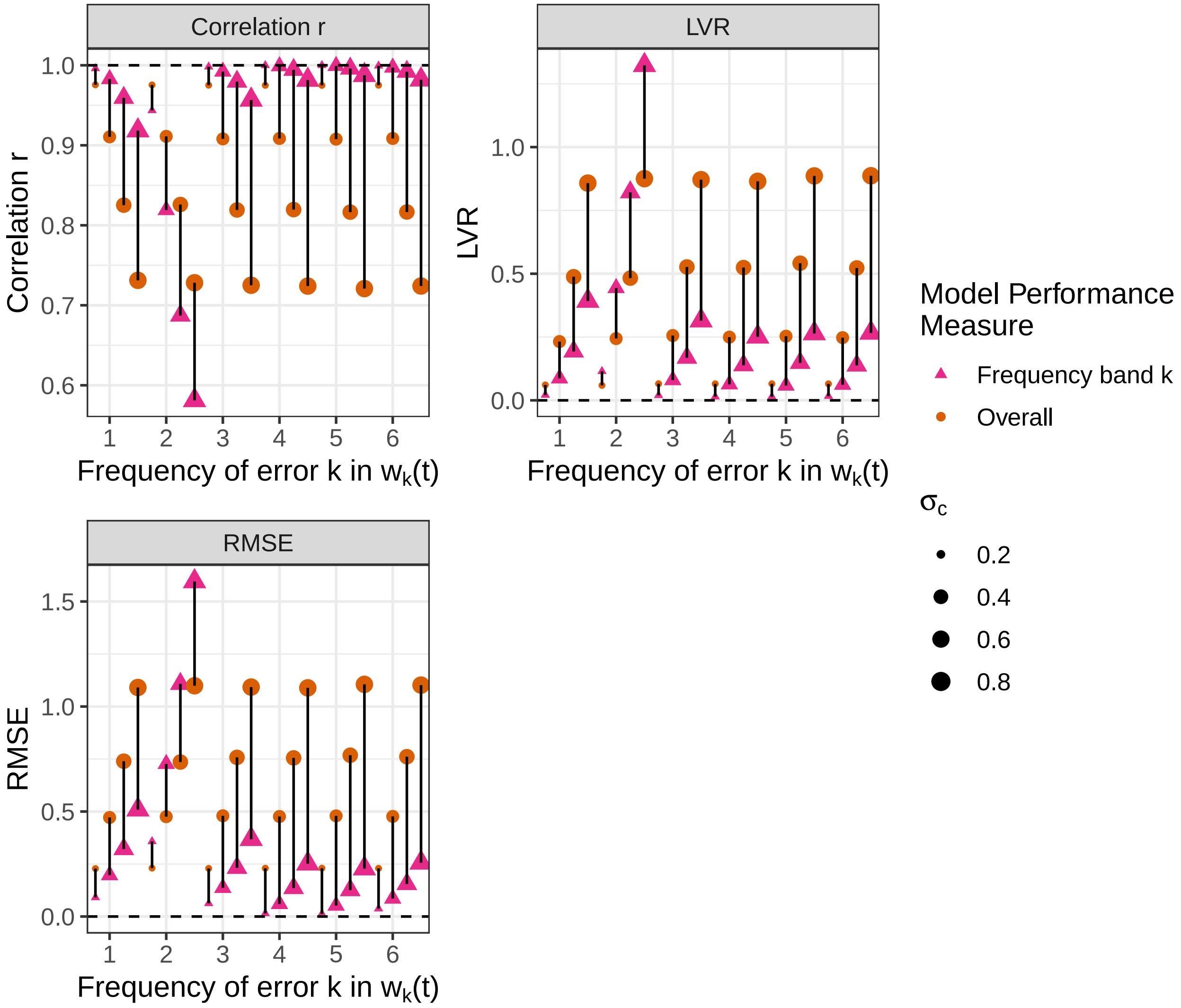

**Figure S2. Comparing simulated observed time series and model predictions using overall model performance (orange circles) and frequency band $k = 6$ model performance (green triangles) for correlation $r$, $RMSE$, and $LVR$ with magnitude of error $\sigma_c = \{0.2, 0.4, 0.6, 0.8\}$. This plot shows the impact of changing the variance of the seasonal component of the simulated observed time series, Var$(x_{(1)}(t)) \in \{1^2, 1.5^2, 2^2\}$.**

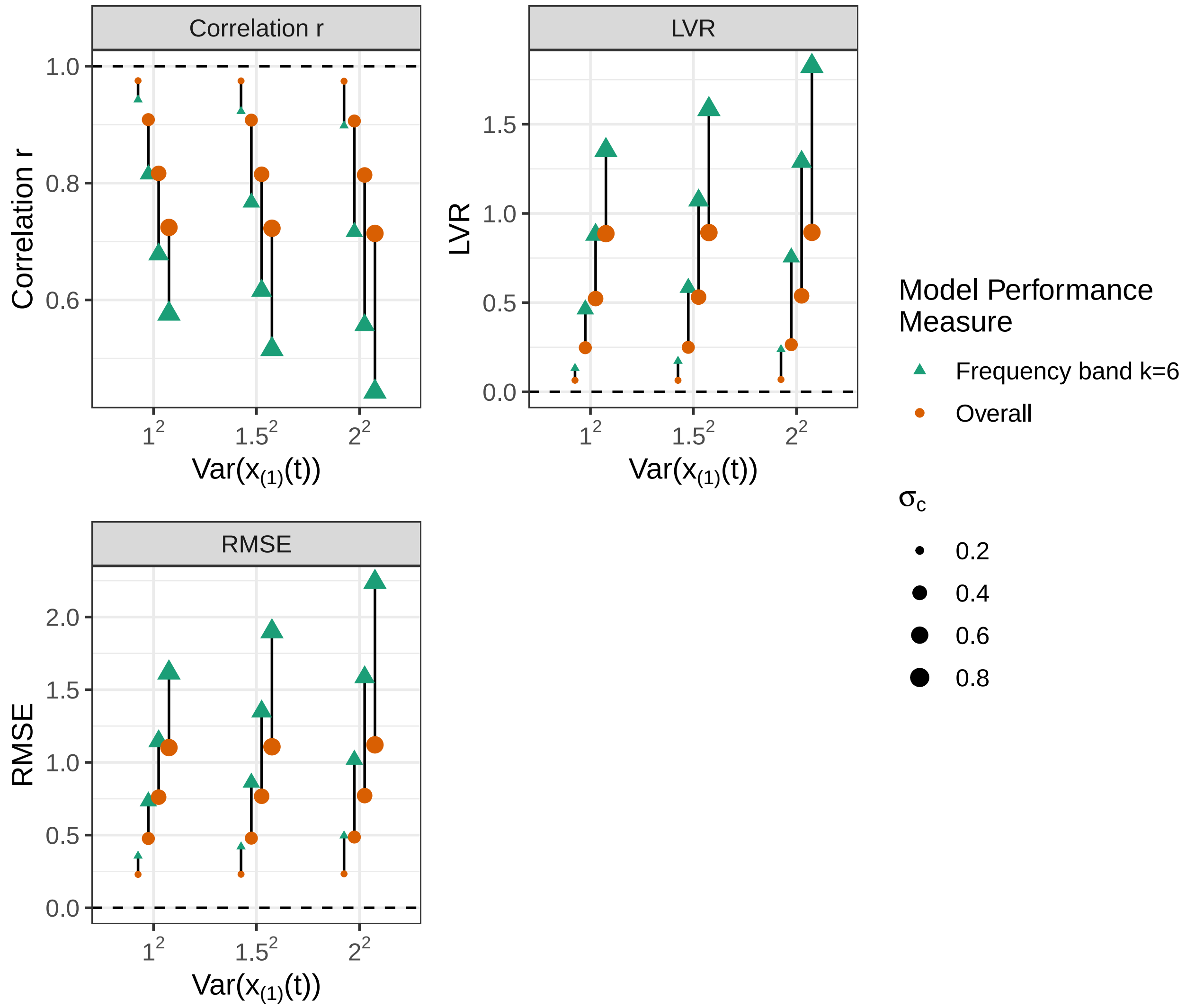

**Figure S3. Map of 17 $PM_{2.5}$ monitors across 8 US cities.**

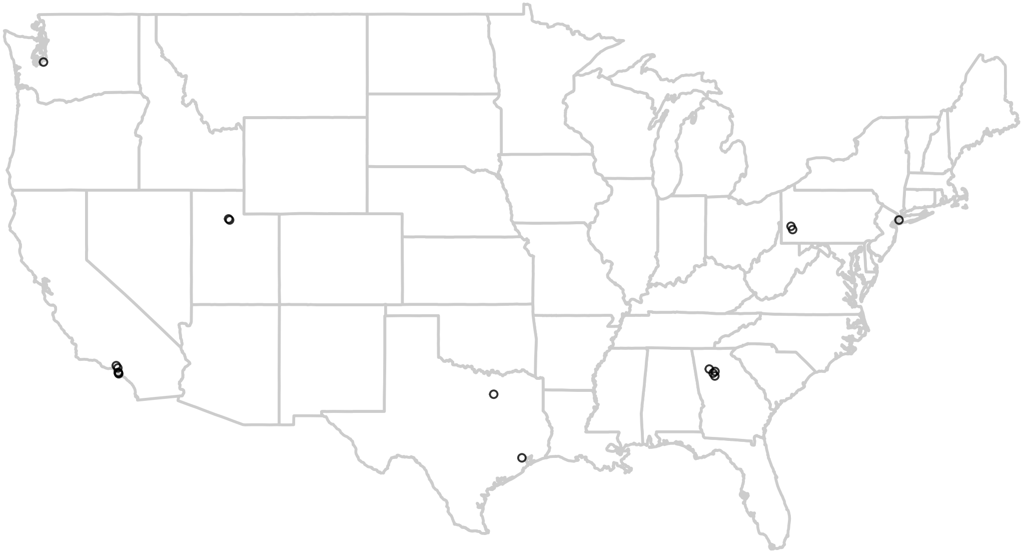

**Table S1. For each monitor ID, table of the corresponding city and state, number of days of data, number and percent of imputed monitor observations, and median (interquartile range) $PM_{2.5}$ ($\mu g/m^3$) for both the monitoring data and FAQSD.**

| Monitor ID | City | State | Days | Imputed *(N (%))* | Monitor *(Median (IQR))* | FAQSD *(Median (IQR))* |
|---|---|---|---|---|---|---|
| 060371002 | Los Angeles | California | 990 | 58 (5.9) | 11.8 (5.3) | 11.16 (5.14) |
| 060371103 | Los Angeles | California | 1422 | 147 (10.3) | 12 (4.97) | 11.93 (5.72) |
| 060374002 | Los Angeles | California | 1527 | 126 (8.3) | 10.3 (4.35) | 10.61 (5.02) |
| 060374004 | Los Angeles | California | 1093 | 46 (4.2) | 9.3 (4.6) | 10.65 (5.05) |
| 060374008 | Los Angeles | California | 1074 | 46 (4.3) | 11.45 (5.1) | 11.12 (5.23) |
| 130670003 | Atlanta | Georgia | 1052 | 58 (5.5) | 10.3 (5.82) | 10.12 (5.79) |
| 130890002 | Atlanta | Georgia | 1051 | 68 (6.5) | 10.13 (5.3) | 10.23 (5.79) |
| 130892001 | Atlanta | Georgia | 686 | 47 (6.9) | 10.7 (5.47) | 10.98 (5.84) |
| 131210032 | Atlanta | Georgia | 686 | 27 (3.9) | 10.5 (5.8) | 11 (5.85) |
| 360810124 | New York | New York | 2351 | 141 (6) | 7 (5.1) | 7.88 (5.91) |
| 420030008 | Pittsburgh | Pennsylvania | 593 | 18 (3) | 9 (5.5) | 10.26 (6.1) |
| 420030064 | Pittsburgh | Pennsylvania | 960 | 53 (5.5) | 13.1 (10.8) | 11.6 (8.64) |
| 481130069 | Dallas | Texas | 1250 | 39 (3.1) | 8.9 (5.4) | 9.5 (5.14) |
| 482011035 | Houston | Texas | 1645 | 72 (4.4) | 11.16 (5.1) | 11.05 (4.82) |
| 490353006 | Salt Lake City | Utah | 958 | 64 (6.7) | 6 (4.32) | 6.24 (4.76) |
| 490353010 | Salt Lake City | Utah | 1003 | 83 (8.3) | 7.2 (5.55) | 6.49 (5.6) |
| 530530029 | Seattle/Tacoma | Washington | 1547 | 106 (6.9) | 5.4 (4.1) | 5.69 (4.6) |

# References

Dominici, Francesca, Aidan McDermott, Scott L Zeger, and Jonathan M Samet. 2003. "Airborne Particulate Matter and Mortality: Timescale Effects in Four Us Cities." *American Journal of Epidemiology* 157 (12): 1055–65.
